# Adjuvant Chemotherapy for Gastric Cancer may Worsen Prognosis in Elderly Women: Retrospective Analysis of Individual Patient Data from the CLASSIC Study

**DOI:** 10.1007/s12029-025-01239-3

**Published:** 2025-06-27

**Authors:** Fumitaka Noji, Hideki Yoshioka, Ryota Jin, Hiroto Hatakeyama, Hiromi Sato, Akihiro Hisaka

**Affiliations:** 1 Clinical Pharmacology and Pharmacometrics, Graduate School of Pharmaceutical Sciences, Chiba University, Chiba, Japan; 2 Clinical Operation, Moderna, Japan; 3 Design and Drug Disposition, Graduate School of Medical and Pharmaceutical Sciences, Chiba University, Chiba, Japan; 4 Present Address: Expert of Modeling and Simulation, Office of Regulatory Science Coordination, Pharmaceuticals and Medical Devices Agency, Tokyo, Japan

**Keywords:** Adjuvant, Capecitabine, Oxaliplatin, Gastric cancer, Individual data, Prognostic factor

## Abstract

**Purpose:**

This research aimed to identify significant prognostic factors that interact with the treatment effect of capecitabine and oxaliplatin (CapeOX), based on individual patient data from the CLASSIC study (NCT00411229), which evaluated the efficacy of adjuvant chemotherapy for gastric cancer.

**Methods:**

Stepwise variable selection of prognostic factors was performed using the Cox proportional hazards model, with patient data from 519 CapeOX-treated and 514 untreated patients.

**Results:**

For all-cause mortality, older women (≥ 55 years) with a serum albumin level ≥ 4.0 g/dL were identified as significant prognostic factors interacting with CapeOX treatment, and unexpectedly, the treatment was associated with poor outcomes in this group. The prognostic significance of serum albumin levels was primarily attributed to the particularly poor survival outcomes observed in untreated patients with serum albumin < 4.0 g/dL. Tumor stage and lymph node status were prognostic factors that interacted with treatment for disease-free survival (DFS). The results showed that in patients with tumor stage (T) ≥ T3 and lymph node metastasis (N) < N2, improvement in DFS with CapeOX was not significant.

**Conclusion:**

As this was a post-hoc analysis, the results should be interpreted as hypothesis-generating rather than definitive. Nevertheless, the findings suggest the need for a more detailed consideration of patient baseline characteristics when determining the adjuvant chemotherapy strategy for gastric cancer.

**Supplementary Information:**

The online version contains supplementary material available at 10.1007/s12029-025-01239-3.

## Introduction

With 19.3 million new cancer cases across 20 world regions in 2020, gastric cancer (GC) is the fifth most frequently diagnosed malignant neoplasm and the fourth leading cause of cancer-related deaths [1–4]. The burden is particularly high in East Asia, where GC accounts for half of all cancer cases and had an estimated mortality rate of 58.3% in 2020 [1]. The National Comprehensive Cancer Network (NCCN) guidelines primarily recommend gastrectomy as standard therapy for patients with stage II and III cancers. Lymph node dissection has been established in Asian countries where the frequency of GC is high, particularly in Japan, and is often performed differently than in other countries. D2 lymphadenectomy has been the standard treatment in Japan. In addition, there are regional differences in systemic chemotherapy strategies that supplement curative surgery in the USA, Europe, and East Asia [5]. In the USA, chemoradiotherapy is widely used based on the INT 0116 trial [6], whereas perioperative chemotherapy is standard in Europe based on the MAGIC [7] and FLOT-4 [8] trials. In East Asia, adjuvant chemotherapy is standard therapy, based on ACTS-GC [9, 10], JACCRO GC-07 [11, 12], and CLASSIC [13, 14]. Capecitabine plus oxaliplatin (CapeOX) has been established as a standard adjuvant chemotherapy for patients with stage II and III GC based on the results of the CLASSIC study. In CLASSIC, CapeOX showed significant improvements in 3-year disease-free survival (DFS) and overall survival (OS) compared to the control group (stratified hazard ratio [HR] 0.58 and 0.66; 95% CI 0.47–0.72 and 0.51–0.85, *p* < 0.0001 and *p* = 0.0015, respectively). On the other hands, 56% of patients in the CapeOX group experienced Grade 3 or 4 adverse events [13, 14].

Personalized treatment approaches that consider a patient’s clinical characteristics have been advocated to optimize treatment outcomes and reduce adverse events. Regional differences in histological type, prevalence of esophagogastric junction cancer, lymph node involvement, and approved drugs may affect the determination of the best treatment strategy [15]. Moreover, sex, age, and disease stage are commonly considered the most important factors in personalized treatment decision-making [16, 17]. However, the incidence of gastric and esophagogastric junction cancer is substantially higher in males [18, 19]. Therefore, treatment strategies may have been optimized based on a male-centric perspective. In the CLASSIC study, sex was not a significant prognostic factor. However, CapeOX significantly improved OS and DFS in males (HRs 0.60 and 0.54; 95% CIs 0.45–0.81 and 0.42–0.70, respectively), while it did not demonstrate significant improvements in OS and DFS in females (HRs 0.93 and 0.71; 95% CIs 0.57–1.51 and 0.48–1.02, respectively) [13, 14]. The incidence of GC has increased rapidly in recent years, reflecting the global aging of the population. Some studies have suggested the advantages of adjuvant chemotherapy in older patients [20–22]. However, older patients remain underrepresented in clinical trials, potentially leading to the adoption of suboptimal treatment modalities. There is also considerable interest in differences in therapeutic efficacy due to potential physiological, pharmacokinetic, and pharmacodynamic differences caused by sex and age [23–27]. These findings highlight the importance of incorporating sex- and age-specific factors into personalized treatment approaches.

To optimize treatment outcomes and reduce adverse events associated with CapeOX, it is important to identify prognostic factors for treatment outcomes, considering patient characteristics such as sex, age, and disease stage severity. Although overall prognostic factors for CapeOX have been reported, interactions between these factors have not yet been fully investigated. Therefore, using individual patient data from the CLASSIC study, we conducted this study to identify prognostic factors, including their important interactions.

## Materials and Methods

### CLASSIC Study and Data Source

The CLASSIC study was an open-label, randomized, phase 3 clinical trial conducted at 35 cancer centers in China, South Korea, and Taiwan (NCT00411229). The study design has been described previously [13, 14]. Eligible patients had histologically confirmed gastric adenocarcinoma and were pathologically staged according to the 6 th edition of the American Joint Committee on Cancer (AJCC)/Union for International Cancer Control (UICC) guidelines, with no evidence of metastatic disease. All patients underwent curative D2 lymphadenectomy within six weeks before randomization and had no macroscopic or microscopic evidence of residual tumor. All patients were ambulatory, aged at least 18 years, and had a Karnofsky Performance Status (KPS) of at least 70. Patients who had previously received chemotherapy, immunotherapy, or radiotherapy for GC were excluded from the study. Anonymized individual patient data from the CLASSIC study were obtained from ClinicalStudyDataRequest.com (CSDR) version 2.0, as of July 24, 2020. The research plan was approved by the Ethical Review Committee of the Graduate School of Pharmaceutical Sciences at Chiba University.

### Statistical Analyses and Multivariate Cox Proportional Hazards Model Analysis

Statistically significant prognostic factors, including interactions with CapeOX, were identified using a stepwise selection method with the PHREG procedure in SAS 9.4. This analysis was based on the Cox proportional hazards model and adjusted for baseline patient characteristics. Baseline characteristics included age, sex, weight, height, BMI, date of surgery, Karnofsky Performance Status, AJCC/UICC stage, tumor stage, lymph node status in the TNM classification, histological type, primary tumor location, and baseline clinical laboratory test results (red blood cell count, platelet count, white blood cell count, neutrophil count, aspartate aminotransferase, alanine aminotransferase, alkaline phosphatase, total bilirubin, direct bilirubin, serum albumin, and creatinine clearance). The combination of sex and age was included as a candidate covariate, given its fundamental role in treatment strategy determination.

To minimize analytical bias, the main effects of the interactions with CapeOX were included in the analysis by setting the PHREG hierarchy option to multiple when the interaction was significant as a covariate. However, this did not necessarily imply a significant main effect. All covariate effects were binarized to address potential nonlinearity. Continuous variables were typically dichotomized at the median. For age, the cutoff was 55 years, which was close to the median in this study (Table 2). When a large number of covariates are systematically searched, the definition of the baseline hazard group in Cox regression analysis changes depending on the covariates adopted. Therefore, the baseline hazard ratio may be less clinically meaningful. For the final model, hazard ratios of the subgroups were calculated relative to the mean of all subjects. Stepwise selection was performed using the PHREG procedure in SAS 9.4, which integrates both forward selection (entry) and backward elimination (removal) to identify significant prognostic factors. Specifically, variables were initially included based on forward selection criteria, and non-significant variables were subsequently removed using backward elimination. This approach ensures optimal model selection while minimizing the risk of overfitting.

Statistical analyses were performed to assess the factors influencing therapeutic efficacy. The evaluation involved using forest plots generated with the R statistical software. Forest plots were created and analyzed using the forestplot package in R. Time-to-event endpoints were analyzed using the Kaplan–Meier survival method. Therapeutic efficacy estimates were calculated as HRs with 95% CIs.

## Results

### Cox Regression Analysis

**Fig. 1:**
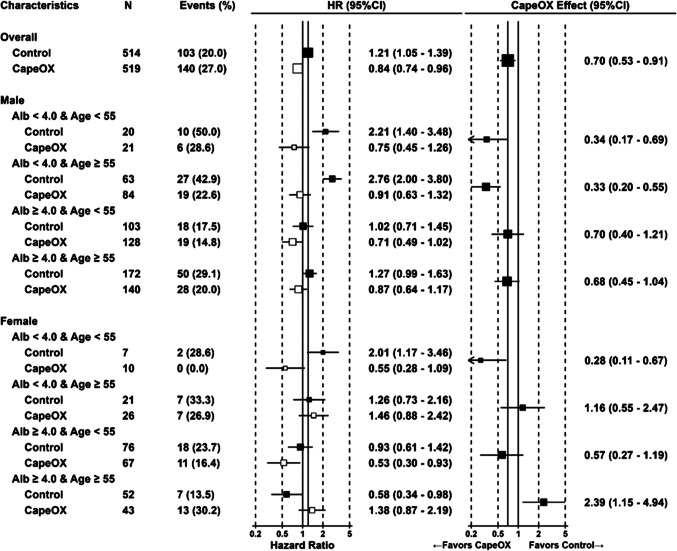
Forest plot of hazard ratios for overall survival in subpopulations classified by factors identified as significant in the Cox regression analysis of CLASSIC study. The hazard ratio to the overall average was presented. The size of each square represents trends in the number of cases

**Fig. 2:**
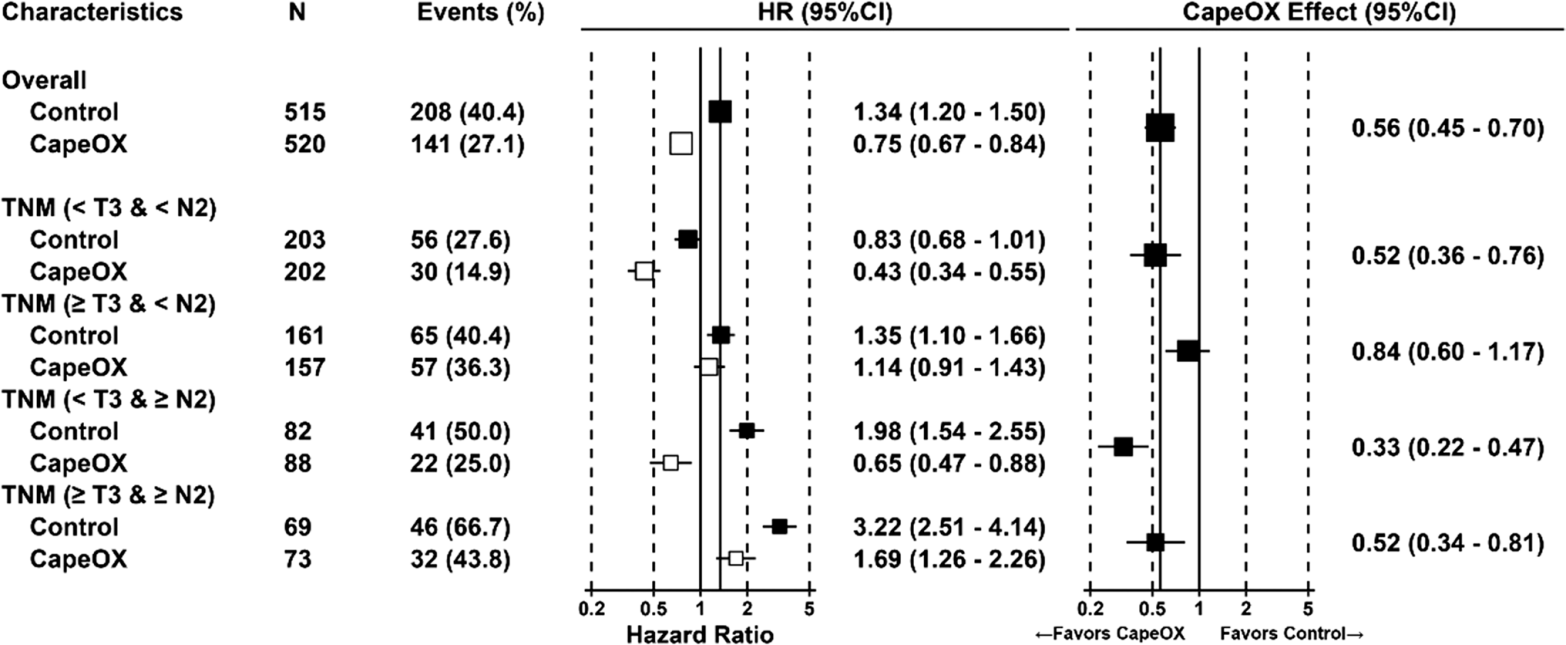
Forest plot of hazard ratios for disease free survival in subpopulations classified by factors identified as significant in the Cox regression analysis of CLASSIC study. The hazard ratio to the overall average was presented. The size of each square represents trends in the number of cases

Prognostic factors associated with adjuvant CapeOX, including their interactions, were identified using individual patient data from the CLASSIC study. Due to missing baseline data, two patients (one in the CapeOX group and one in the control group) were excluded. As a result of stepwise selection with Cox proportional hazards model analysis, serum albumin and the combination of sex and age (females aged ≥ 55 years) were identified as significant interaction factors with CapeOX (*p* < 0.05, Table 1A). AJCC/UICC stage, serum albumin level, CapeOX treatment, tumor stage, and BMI were identified as significant independent prognostic factors for OS. Tumor stage and nodal status were identified as significant interaction factors with CapeOX, and tumor stage, nodal status, CapeOX treatment, and age were identified as significant independent prognostic factors for DFS (*p* < 0.05, Table 1B). Based on Cox regression analysis, changes in the therapeutic efficacy of CapeOX for each subgroup are presented as forest plots (Figs. 1 and 2).

**Table 1:** Significant prognostic factors identified by forward–backward stepwise selection of the Cox proportional hazards model analysis (*p* < 0.05)

A. Overall survival			
**Objective**	**Parameter estimate**	**Standard error**	**P value**
AJCC/UICC Stage ≥ III	0.750	0.160	< 0.0001
Albumin ≥ 4.0 g/dL	−0.775	0.187	< 0.0001
CapeOX	−1.079	0.362	0.0028
CapeOX *Albumin ≥ 4.0 g/dL	0.720	0.288	0.013
CapeOX * Female * Age ≥ 55	1.461	0.612	0.017
Tumor stage ≥ T3	0.347	0.151	0.022
BMI ≥ Median(21.6)	−0.286	0.133	0.031
Female * Age ≥ 55 ψ	−0.693	0.416	0.095
Age ≥ 55 ψ	0.223	0.223	0.315
CapeOX/Female ψ	−0.208	0.467	0.656
CapeOX/Age ≥ 55 ψ	−0.024	0.337	0.944
**B. Disease free survival**			
Nodal status ≥ N2	0.868	0.141	< 0.0001
Tumor stage ≥ T3	0.486	0.139	0.0005
CapeOX	−0.648	0.192	0.0007
Age ≥ 55	0.281	0.111	0.012
CapeOX * Tumor stage ≥ T3	0.476	0.223	0.033
CapeOX * Nodal status ≥ N2	−0.473	0.223	0.034

ψ Not significant alone but shown because these were included as significant interaction prognostic factors

CapeOX showed significant survival efficacy in males and females aged < 55 with serum albumin < 4.0 g/dL. Conversely, CapeOX was associated with poor outcomes in females aged ≥ 55 years with serum albumin ≥ 4.0 g/dL (HR 2.39; 95% CI, 1.15–4.94). For females aged ≥ 55 years with serum albumin < 4.0 g/dL, the therapeutic efficacy of CapeOX was not significant (HR 1.16; 95% CI, 0.55–2.47). CapeOX was more beneficial in patients with tumor stage no greater than T3 and nodal status N2 or greater in DFS compared with the overall population (HR 0.56, 95%CI 0.45–0.70), whereas no significant difference in DFS was observed in patients with tumor stage T3 or greater and nodal status no greater than N2 (HR 0.84; 95% CI, 0.60–1.17). Patient characteristics in each group, classified according to the selected factors, were judged to be appropriately balanced (Tables 2, S1, and S2).

**Table 2:** Distribution of patient characteristics classified by significant prognostic factors identified through forward–backward stepwise selection of the Cox proportional hazards model analysis

Category	Characteristics	CapeOX (*n* = 519)		Control (*n* = 514)	
		n	%	n	%
Sex	Female	146	(28.1%)	156	(30.4%)
	Male	373	(71.9%)	358	(69.6%)
Age	Median		56.1 y		55.8 y
	≥ 55	293	(56.5%)	308	(59.9%)
	< 55	226	(43.5%)	206	(40.1%)
Age & Sex	Female & Age ≥ 55	69	(13.3%)	73	(14.2%)
	Female & Age < 55	77	(14.8%)	83	(16.1%)
	Male & Age ≥ 55	224	(43.2%)	235	(45.7%)
	Male & Age < 55	149	(28.7%)	123	(23.9%)
Albumin (g/mL)	High (≥ 4.0 g/dL)	378	(72.8%)	403	(78.4%)
	Low (< 4.0 g/dL)	141	(27.2%)	111	(21.6%)
Tumor stage	≥ T3	229	(44.1%)	230	(44.7%)
	< T3	290	(55.9%)	284	(55.3%)
Nodal status	≥ N2	161	(31.0%)	151	(29.4%)
	< N2	358	(69.0%)	363	(70.6%)
AJCC/UICC Stage	≥ III	265	(51.1%)	253	(49.2%)
	< III	254	(48.9%)	261	(50.8%)
BMI	≥ Median(21.6)	249	(48.0%)	262	(51.0%)
	< Median(21.6)	270	(52.0%)	252	(49.0%)

### Kaplan–Meier curves

Kaplan–Meier curves for each identified factor were generated and compared with the results of the Cox regression analysis (Supplementary Fig. 1). Kaplan–Meier curves for males of all ages and females aged < 55 years (Supplementary Fig. 1b, c) showed a reduction in risk with CapeOX. In contrast, curves for females aged ≥ 55 years (Supplementary Fig. 1c) showed an increased risk. Regarding serum albumin levels, the therapeutic efficacy of CapeOX was weaker in patients with serum albumin < 4.0 g/dL (Supplementary Fig. 1d). Kaplan–Meier curves for DFS were also compared by tumor stage and nodal status, and the degree of therapeutic efficacy was attenuated in patients with tumor stage T3 or greater and nodal status no greater than N2 (Supplementary Fig. 2), consistent with the results of the Cox regression analysis. The Kaplan–Meier curves showed no problematic crossings between the CapeOX and control groups, suggesting that the proportional hazards assumption of the Cox analysis was maintained.

As different interaction prognostic factors were identified between OS and DFS, we confirmed the consistency of therapeutic efficacy based on the Kaplan–Meier curves. For both OS and DFS, therapeutic efficacy tended to be weaker in females aged ≥ 55 than in females aged < 55 (Figure S3). Additionally, patients with serum albumin < 4.0 g/dL showed better treatment outcomes than those with serum albumin ≥ 4.0 g/dL. Similarly, the benefits of treatment in terms of both OS and DFS were unclear for patients with tumor stage T3 or greater and nodal status less than N2 nodal status (Figure. S4). Overall, although the changes that reached statistical significance differed between OS and DFS, the trends were similar for both outcomes.

### Adverse Events and Treatment Course

Cox analysis revealed that sex, age, tumor stage, and nodal status affected OS or DFS. The incidence of adverse events and dose modifications classified according to these factors are summarized in Table 3. Neutropenia, decreased appetite, and peripheral neuropathy tended to be more frequent in females aged ≥55 years compared to other groups. In addition, females aged ≥55 years tended to have lower cycle completion rates, more frequent dose reductions, and more dose interruptions.

**Table 3:** Comparison of number of patients for adverse events or treatment modifications summary

(A) Adverse events reported > 40% incidence, classified by sex and age								
Adverse events	Male & Age < 55		Male & Age ≥ 55		Female & Age < 55		Female & Age ≥ 55	
	n	%	n	%	n	%	n	%
Nausea	98	65.8%	122	54.5%	59	76.6%	48	69.6%
Neutropenia	83	55.7%	124	55.4%	47	61.0%	46	66.7%
Decreased appetite	78	52.3%	126	56.3%	39	50.6%	51	73.9%
Neuropathy peripheral	76	51.0%	114	50.9%	42	54.5%	45	65.2%
Diarrhea	66	44.3%	99	44.2%	40	51.9%	30	43.5%
Vomiting	43	28.9%	66	29.5%	45	58.4%	36	52.2%
(B) Summary of the treatment course and modifications classified by sex and age								
Treatment course & modifications	Male & Age < 55		Male & Age ≥ 55		Female & Age < 55		Female & Age ≥ 55	
	n	%	n	%	n	%	n	%
Cycle completion	110	73.8%	141	62.9%	56	72.7%	39	56.5%
Dose reduction	52	34.9%	92	41.1%	41	53.2%	36	52.2%
Treatment interruption	43	28.9%	116	51.8%	24	31.2%	36	52.2%
Relative dose intensity (≥ 70)	122	81.9%	182	81.3%	60	77.9%	54	78.3%
(C) Adverse events reported with > 40% incidence classified by tumor stage (T) and nodal status (N)								
Adverse events	≥ T3 & ≥ N2		≥ T3 & < N2		< T3 & ≥ N2		< T3 & < N2	
	n	%	n	%	n	%	n	%
Nausea	43	58.9%	92	59.0%	62	70.5%	129	63.9%
Neutropenia	47	64.4%	88	56.4%	49	55.7%	116	57.4%
Decreased appetite	41	56.2%	85	54.5%	49	55.7%	119	58.9%
Neuropathy peripheral	33	45.2%	80	51.3%	50	56.8%	114	56.4%
Diarrhea	36	49.3%	65	41.7%	36	40.9%	98	48.5%
Vomiting	22	30.1%	58	37.2%	31	35.2%	79	39.1%
(D) Treatment course and modification summary classified according to the tumor stage (T) and nodal status (N)								
Treatment course & modifications	≥T3 & ≥N2		≥T3 & <N2		<T3 & ≥N2		<T3 & < N2	
	n	％	n	％	n	％	n	％
Cycle completion	44	60.3%	94	60.3%	61	69.3%	147	72.8%
Dose reduction	34	46.6%	67	42.9%	35	39.8%	85	42.1%
Treatment interruption	28	38.4%	64	41.0%	42	47.7%	85	42.1%
Relative dose intensity (≥ 70)	62	84.9%	123	78.8%	69	78.4%	163	80.7%

^*^Patients who did not start CapeOX treatment was counted as"NO"

## Discussion

### The Effect of Age and Sex

To our knowledge, this study is the first to systematically explore interacting prognostic factors, including sex and age, that affect the therapeutic efficacy of adjuvant chemotherapy in stage II/III GC. The pre-planned subgroup analysis of the CLASSIC study showed that CapeOX was significantly beneficial compared to controls in males, but the benefit was not statistically significant in females [13, 14]. Our study consistently suggested that CapeOX provides a survival benefit in females aged < 55 years and in all males. However, no survival benefit was observed in females aged ≥ 55 years. Moreover, when stratified by age and serum albumin in addition to sex, the results showed that prognosis was statistically significantly worse (*p* < 0.05) for female patients aged ≥ 55 years with serum albumin ≥ 4.0 g/dL when treated with CapeOX (Fig. 1). Given that the sample size for this subgroup was relatively small (43 and 52 patients in the treatment and control groups, respectively), and that this was a post-hoc analysis of a single clinical study, the findings should be interpreted hypothetically, considering potential confounding factors. Nevertheless, regarding age, a subgroup analysis of ACTS-GC also suggested that the therapeutic efficacy of S-1 was lower for older patients in terms of OS [9, 10]. In JACCRO GC-07, the therapeutic efficacy of the combination of S-1 and docetaxel was lower in females than in males for both relapse-free survival (RFS) and OS [11, 12]. In ACT-GC and JACCRO GC-07, however, interactions between sex, age, and treatment were not investigated. Furthermore, the number of female participants generally tends to be lower in GC clinical studies, potentially leading to male-centered therapy optimization. Considering our findings, along with previous studies, it is essential to assess the therapeutic efficacy of adjuvant chemotherapy for GC, specifically focusing on older females to optimize personalized treatment strategies.

Our analysis showed that females aged ≥ 55 years had higher rates of dose reductions, dose interruptions, and a lower cycle completion rate (56.5%; Table 3b). The original CLASSIC study reported that receiving more than six treatment cycles was important for improving outcomes. The J-CLASSIC study also reported that cycle completion rates were lower in females (72%) than in males (79%) [28]. In the JCOG1104 study, the number of cycles of adjuvant S-1 chemotherapy (8 vs. 4 cycles) was compared in patients with pathological stage II GC after curative resection, and it was found that the 8-cycle group had a better prognosis than the 4-cycle group [29]. The hazard ratio (HR) for recurrence-free survival was 2.52 (95% CI, 1.11–5.77), suggesting the need for long-term adjuvant chemotherapy. The cumulative total dose of S-1 in adjuvant chemotherapy has also been reported to affect long-term outcomes after radical gastrectomy for GC [30].

In this study, the incidence of some adverse events was higher in females aged ≥ 55 years than in other groups (Table 3a). In particular, decreased appetite was significantly more frequent in females aged ≥ 55 years (73.9%) compared with the other groups, which may have contributed to dose reductions, treatment interruptions, and lower completion rates. Decreased appetite may lead to weight loss, and it has been reported that the rate of weight loss after gastrectomy affects the continuation of adjuvant chemotherapy and prognosis [31, 32]. Therefore, managing decreased appetite and weight loss through aggressive nutritional support may be particularly important for older females.

Traditionally, in cancer therapy, the dose of cytotoxic anticancer agents is determined by body surface area (BSA). However, in the era of precision medicine, personalized treatment strategies should be considered. Dose adjustment based on renal function, creatinine clearance, and other parameters is widely used to optimize pharmacokinetics. However, dose adjustment based on hepatic function is rarely performed, except in specific conditions, such as liver failure, due to the lack of an appropriate marker. A 0.8% decrease in hepatic drug clearance per year has been reported in adults over 40 years of age [33]; this change is particularly important in older patients and should be considered in future dose adjustments. CapeOX is administered at an initial dose determined by BSA, which may have led to overdosing in females aged ≥ 55 years, resulting in a higher incidence of adverse events. For females aged ≥ 55 years, it may be an option to start at a lower dose and aim for treatment completion, taking into account the patient's sex and age.

Microsatellite instability (MSI) status has been recognized as a clinical decision-making factor [34–38] and may be useful for predicting the therapeutic efficacy of 5-FU-based chemotherapy for stage II and III GC [39]. High MSI status correlates with mismatch repair deficiency (dMMR), which is more common in older women [40]. HER2 overexpression in patients with GC is another poor prognostic factor [41]. However, this analysis did not include information on MSI, dMMR, and HER2 status because these data were unavailable in the CLASSIC study.

### The Effect of Serum Albumin

Patients with low serum albumin levels tended to show greater therapeutic efficacy for OS (Fig. 1). Conversely, in patients with high serum albumin levels, survival was higher among those who did not receive adjuvant chemotherapy, while CapeOX had little impact on their survival. These findings suggest that low serum albumin levels may weaken cancer resistance, making adjuvant chemotherapy more beneficial, particularly for patients who would otherwise not receive adjuvant treatment.

### The Effect of Tumor Stage and Lymph Nodal Status

Various clinical trials on adjuvant chemotherapy for GC have been reported, including subgroup analyses based on tumor stage and nodal status. In the subgroup analysis of the CLASSIC study, the HR was 0.72 (95% CI: 0.54–0.95) for patients with tumor stage T3 or greater, and 0.46 (95% CI: 0.33–0.65) for patients with tumor stage no greater than T3, showing a trend toward decreased therapeutic efficacy in more advanced tumor stages. In contrast, in the ACTS-GC study, the HRs for T2 and T3 tumor stages were 0.652 (95% CI: 0.471–0.902) and 0.690 (95% CI: 0.511–0.932), respectively [9, 10], suggesting similar therapeutic efficacy regardless of tumor stage. In the CLASSIC study, the HRs were 0.57 (95% CI: 0.46–0.72) for patients with N2 or greater nodal status and 0.79 (95% CI: 0.39–1.60) for those with no more than N1 nodal status. Although the confidence intervals were wide and difficult to interpret, therapeutic efficacy tended to decrease with more advanced nodal status. In ACTS-GC, the HRs were 0.317 (95% CI: 0.127–0.790) for N0, 0.608 (95% CI: 0.440–0.840) for N1, and 0.839 (95% CI: 0.612–1.150) for N2, but the therapeutic efficacy in N2 patients remained unclear. On the other hand, the subgroup analysis of JACCRO GC-07 [11, 12] showed HRs of 1.33 (95% CI: 0.375–4.718) for N1, 0.514 (95% CI: 0.289–0.913) for N2, and 0.642 (95% CI: 0.465–0.887) for N3, indicating that the relationship between nodal status and therapeutic efficacy was not clearly defined. Thus, the association between the therapeutic efficacy of adjuvant chemotherapy for GC and nodal status has not been clearly established, with varying results across trials.

In the CLASSIC study, there were only 103 N0 patients out of a total of 1035, and the N1 threshold may not have been sufficiently powerful to detect an effect. Therefore, in the present study, the cutoff value for nodal status was set at N2, and the analysis was performed considering the interaction between tumor stage and nodal status. This result suggests that the therapeutic efficacy of CapeOX may be reduced in patients with tumor stage no greater than T3 and nodal status of N2 or greater in terms of DFS. The impact of these factors on prognosis should be considered separately. Figure 2 shows that the prognosis of the control group tended to worsen with the progression of tumor stage or nodal status, consistent with the general understanding that survival differences between patients within the same stage should be small. However, the prognosis did not vary significantly with nodal status progression, which could be interpreted as adjuvant chemotherapy effectively mitigating the risk associated with more advanced nodal involvement. As a result, the benefit of adjuvant chemotherapy may not have been fully demonstrated in patients with advanced disease but rather in those with limited nodal involvement. Therefore, the findings of this research suggest the need for further investigation into treatment strategies based not only on AJCC/UICC staging but also on detailed assessments of tumor stage and nodal status. Moreover, there is a strong case for the future development of personalized treatment strategies to better select patients likely to benefit from adjuvant chemotherapy with CapeOX.

### Limitations

First, this was a retrospective post-hoc analysis, and our findings should be considered hypothesis-generating rather than definitive conclusions. Therefore, confirmation of these results in a prospective study is required. Second, the median follow-up period was 62.4 months in the treatment group and 62.6 months in the control group at the time of the final analysis. Many cases had no confirmed events, resulting in potential immortal time bias and immature data. Third, the number of female patients included in the study was relatively small, and when further stratified by age (e.g., ≥ 55 years), the sample size became even smaller. This limits the generalizability of our findings, particularly for older female patients.

## Conclusions

This study identified key prognostic factors for CapeOX adjuvant chemotherapy in GC, including interactions between age, sex, and disease status, based on the CLASSIC study. Notably, it was suggested that females aged ≥ 55 years may not benefit from CapeOX in terms of overall survival (OS). Moreover, the therapeutic efficacy of CapeOX may be attenuated by disease stage and nodal status. These results suggest that treatment strategies should consider age, sex, and patient condition. In addition, close monitoring of adverse events is important to ensure treatment completion. These findings highlight the need for a prospective clinical study to confirm our results, particularly in females aged ≥ 55 years. Treatment strategies for stage II and III GC differ across regions. Still, these findings may be applicable not only in East Asia but also in non-Asian countries when considering individualized treatment strategies, as the prognostic factors identified were based on fundamental clinical patient information.

In conclusion, this study underscores the importance of considering various patient factors when planning adjuvant chemotherapy for GC with CapeOX.

## Supplementary Information

Below is the link to the electronic supplementary material.

Supplementary file1 (DOCX 1210 KB)

## Data Availability

No datasets were generated or analysed during the current study.
