# Supplementary material for: Adjuvant Chemotherapy for Gastric Cancer may Worsen Prognosis in Elderly Women: Retrospective Analysis of Individual Patient Data from the CLASSIC Study
Source: J Gastrointest Cancer. 2025 Jun 27;56(1):142. doi: 10.1007/s12029-025-01239-3 (PMC12202623; doi:10.1007/s12029-025-01239-3)
Supplement: Supplementary file 1 — Supplementary file1 (DOCX 1210 KB) [file 12029_2025_1239_MOESM1_ESM.docx]

Adjuvant chemotherapy for gastric cancer may worsen prognosis in elderly women: Retrospective analysis of individual patient data from CLASSIC trial

**Supplemental appendix**

Table S1. Patient characteristic for all variables which were used for Cox Proportional Hazards model.

|  |  | **Capecitabine**  **and oxaliplatin  (n=519)** | | |  | **Surgery only  (n=514)** | | |
| --- | --- | --- | --- | --- | --- | --- | --- | --- |
| Sex | Female | 146 |  | 28.1% |  | 156 |  | 30.4% |
| Age | Median | 56.1 y | | |  | 55.8 y | | |
|  | >=55 | 293 |  | 56.5% |  | 308 |  | 59.9% |
| Age & Sex | Female & Age >=55 | 69 |  | 13.3% |  | 73 |  | 14.2% |
|  | Female & Age <55 | 77 |  | 14.8% |  | 83 |  | 16.1% |
|  | Male & Age >=55 | 224 |  | 43.2% |  | 235 |  | 45.7% |
|  | Male & Age <55 | 149 |  | 28.7% |  | 123 |  | 23.9% |
| Albumin (g/mL) | ≧ 4.0 | 378 |  | 72.8% |  | 403 |  | 78.4% |
| Tumour stage | ≧ T3 | 229 |  | 44.1% |  | 230 |  | 44.7% |
| Nodal status | ≧ N2 | 161 |  | 31.0% |  | 151 |  | 29.4% |
| AJCC/UICC Stage | ≧ III | 265 |  | 51.1% |  | 254 |  | 49.4% |
| BMI | ≧ Median（21.6） | 249 |  | 48.0% |  | 262 |  | 51.0% |
| Weight | ≧ Median（57.0） | 241 |  | 46.4% |  | 238 |  | 46.3% |
| Height | ≧ Median（1.64） | 256 |  | 49.3% |  | 268 |  | 52.1% |
| Date from surgery (days) | ≧ Median（35） | 240 |  | 46.2% |  | 292 |  | 56.8% |
| Karnofsky performance | ≧ 90 | 158 |  | 30.4% |  | 170 |  | 33.1% |
| Complication | Yes | 219 |  | 42.2% |  | 209 |  | 40.7% |
| primary tumor location | ANTRUM | 236 |  | 45.5% |  | 233 |  | 45.3% |
| HGB (g/mL) | ≧ 10 | 489 |  | 94.2% |  | 482 |  | 92.9% |
| Ccr (mL/min) | ≧ median | 226 |  | 43.5% |  | 255 |  | 49.1% |
| RBC (×100,000 /μL) | ≧ 4 | 335 |  | 64.5% |  | 332 |  | 64.0% |
| PLT ( /μL) | ≧ 150000 | 497 |  | 95.8% |  | 494 |  | 95.2% |
| WBC( /μL) | ≧ 4000 | 483 |  | 93.1% |  | 475 |  | 91.5% |
| AST (IU/L) | ≧ 40 | 21 |  | 4.0% |  | 16 |  | 3.1% |
| ALT (IU/L) | ≧ 40 | 48 |  | 9.2% |  | 43 |  | 8.3% |
| ALP (IU/L) | ≧ 100 | 114 |  | 22.0% |  | 120 |  | 23.1% |
| Total Bilirubin (mg/dL) | ≧ 1 .0 | 30 |  | 5.8% |  | 24 |  | 4.6% |
| Direct Bilirubin (mg/dL) | ≧ 0.4 | 27 |  | 5.2% |  | 14 |  | 2.7% |
| Albumin (g/dL) | ≧ 4.0 | 378 |  | 72.8% |  | 403 |  | 77.6% |
| RDI | ≧ 70 | 418 |  | 80.4% |  | - |  |  |
| Cycle completion | Yes | 346 |  | 66.5% |  | - |  |  |
| Dose reduction | Yes | 221 |  | 42.5% |  | - |  |  |
| Interruption | Yes | 219 |  | 42.1% |  | - |  |  |
| AE Nausea | Yes | 327 |  | 62.9% |  | 22 |  | 4.3% |
| AE Neutropenia | Yes | 301 |  | 57.9% |  | 5 |  | 1.0% |
| AE Decreased appetite | Yes | 295 |  | 56.7% |  | 20 |  | 3.9% |
| AE Neuropathy peripheral | Yes | 278 |  | 53.5% |  | 2 |  | 0.4% |
| AE Diarrhea | Yes | 235 |  | 45.2% |  | 54 |  | 10.5% |
| AE Vomitting | Yes | 190 |  | 36.5% |  | 18 |  | 3.5% |
| AE Fatigue | Yes | 157 |  | 30.2% |  | 12 |  | 2.3% |
| AE Thrombocytopenia | Yes | 131 |  | 25.2% |  | 1 |  | 0.2% |

Table S2. Patient characteristic by Sex and Age (cut off : 55 years).

In the chi-square test conducted across eight groups, no significant differences were observed for any factors except BMI. For the factor of BMI, especially within the subgroup of females aged below 55 in the surgery-only group, a significant difference was detected when compared with the other seven groups.

|  |  | Capecitabine and oxaliplatin (n=519) | | | | | | | | | | | |  | Surgery only (n=514) | | | | | | | | | | | |
| --- | --- | --- | --- | --- | --- | --- | --- | --- | --- | --- | --- | --- | --- | --- | --- | --- | --- | --- | --- | --- | --- | --- | --- | --- | --- | --- |
|  |  | Female & Age >=55 (n=69) | | | Female & Age <55 (n=77) | | | Male & Age >=55 (n=224) | | | Male & Age <55 (n=149) | | |  | Female & Age >=55 (n=73) | | | Female & Age <55 (n=83) | | | Male & Age >=55 (n=235) | | | Male & Age <55 (n=123) | | |
| Albumin (g/mL) | ≧ 4.0 | 43 |  | (62%) | 67 |  | (87%) | 140 |  | (63%) | 128 |  | (86%) |  | 52 |  | (71%) | 76 |  | (92%) | 172 |  | (73%) | 103 |  | (84%) |
| Tumour stage | ≧ T3 | 31 |  | (45%) | 41 |  | (53%) | 95 |  | (42%) | 62 |  | (42%) |  | 37 |  | (51%) | 48 |  | (58%) | 100 |  | (43%) | 45 |  | (37%) |
| Nodal status | ≧ N2 | 20 |  | (29%) | 23 |  | (30%) | 72 |  | (32%) | 46 |  | (31%) |  | 23 |  | (32%) | 28 |  | (34%) | 67 |  | (29%) | 33 |  | (27%) |
| AJCC/UICC Stage | ≧ III | 34 |  | (49%) | 41 |  | (53%) | 119 |  | (53%) | 71 |  | (48%) |  | 40 |  | (55%) | 44 |  | (53%) | 113 |  | (48%) | 57 |  | (46%) |
| BMI | ≧ Median（21.6） | 37 |  | (54%) | 30 |  | (39%) | 108 |  | (48%) | 74 |  | (50%) |  | 45 |  | (62%) | 28 |  | (34%) | 119 |  | (51%) | 70 |  | (57%) |

Figure S1. Kaplan-Meier curves for OS by significant interacting factors identified by the Cox proportional hazards model analysis. (A) Intent-to-treat population, (B) Male, (C) Female, (D) Stratified by albumin level (cutoff value is 4.0 g/dL). Numbers arranged as a table in each plot represents the number of patients evaluated at each observation. Censoring indicators are omitted for visual clarity.


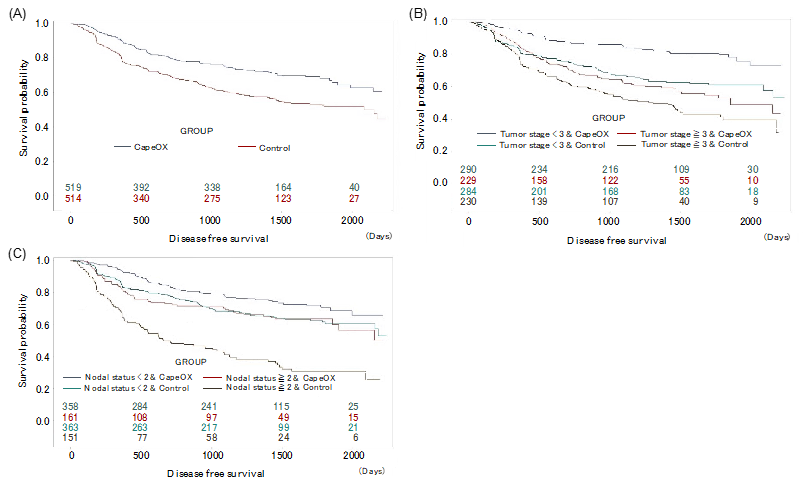


Figure S2. Kaplan-Meier curves for DFS by significant interacting factors which identified by the Cox proportional hazards model analysis. (A) Intent-to-treat population, (B) Stratified by tumor stage, (C) Stratified by nodal status. Numbers arranged as a table in each plot represents the number of patients evaluated at each observation. Censoring indicators are omitted for visual clarity.

(a) by treatment and age in female (b) by treatment and age in male


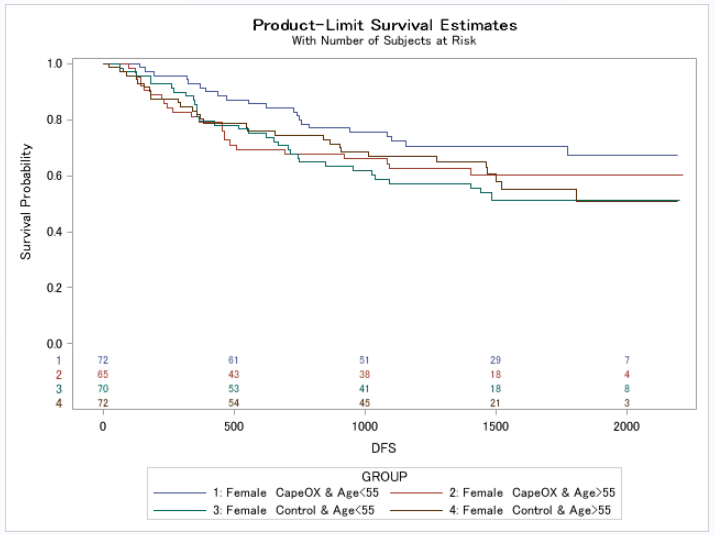

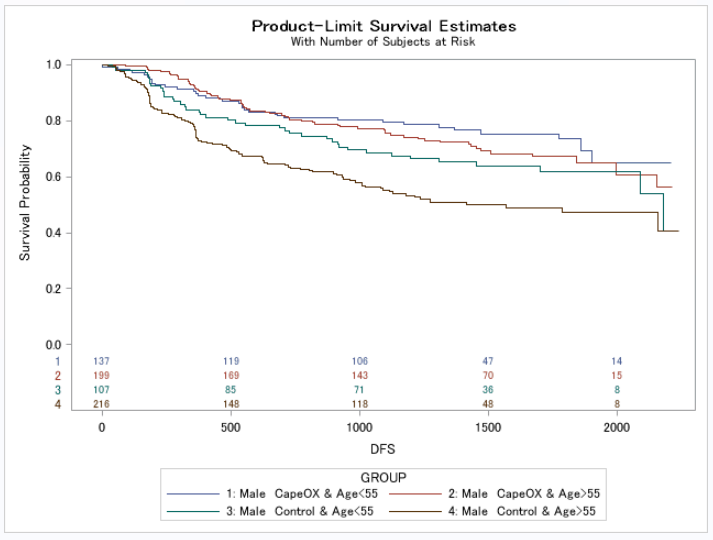


(c) by treatment and baseline albumin value (cut-off 4.0g/dL).


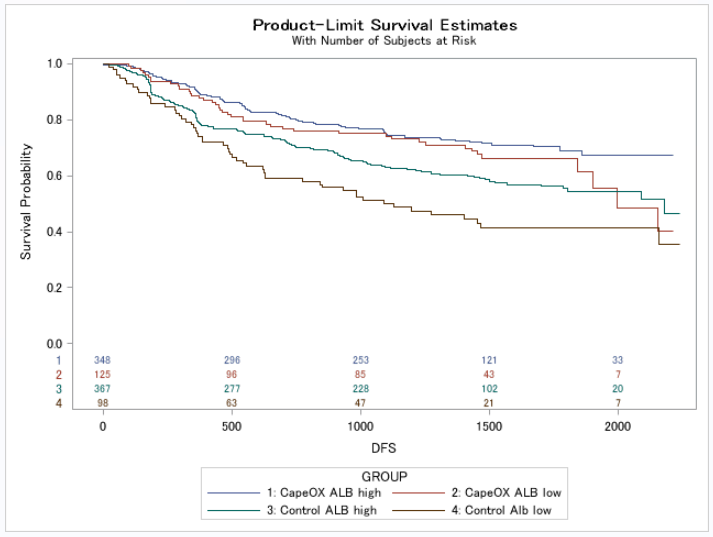


Figure S3. Kaplan-Meier curve of Disease free survival stratified by the significant interactions factors selected by stepwise selection method of Cox Proportional Hazards model in overall survival (a) by treatment and age in female (b) by treatment and age in male (c) by treatment and baseline albumin value (cut-off 4.0g/dL). The trends of the Kaplan-Meier curves of disease free survival are consist with the Kaplan-Meier curves of overall survival.

Note: Censoring indicators are not shown in the Kaplan-Meier curve for clarity.

(a) by treatment and tumor stage (b) by treatment and nodal status.


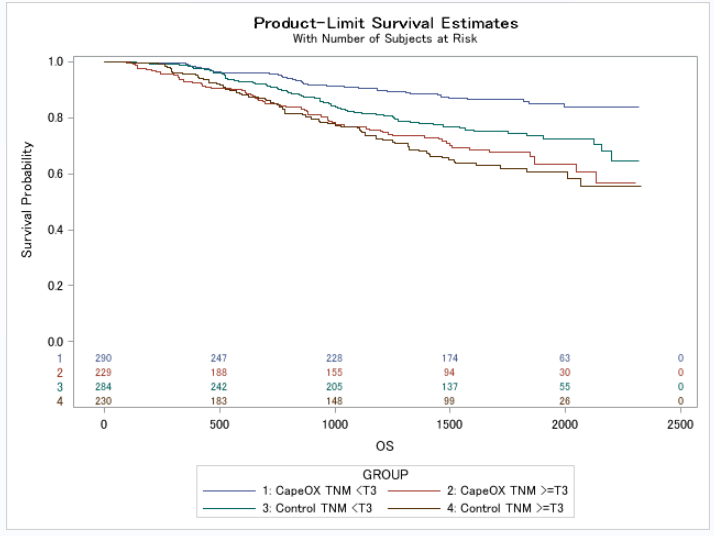

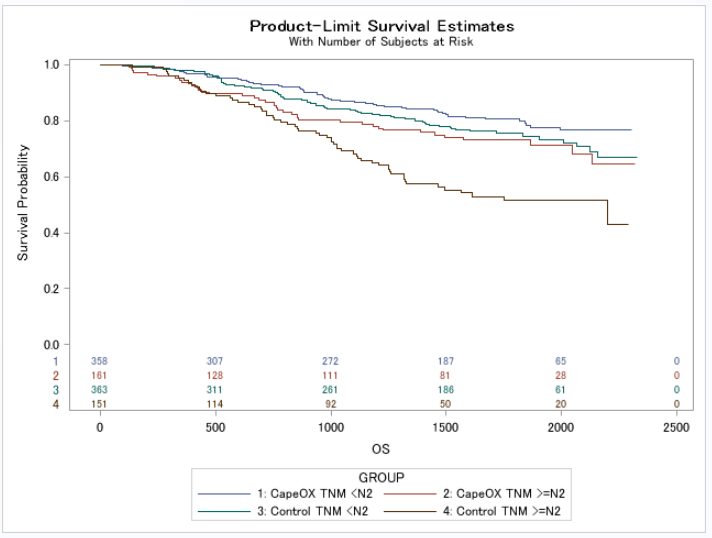


Figure S4. Kaplan-Meier curve of Overall survival stratified by the significant interactions factors selected by stepwise selection method of Cox Proportional Hazards model in disease free survival (a) by treatment and tumor stage (b) by treatment and nodal status. The trends of the Kaplan-Meier curves of disease free survival are consist with the Kaplan-Meier curves of overall survival. Note: Censoring indicators are not shown in the Kaplan-Meier curve for clarity.
